# The effects of multi mineral-vitamin D and vitamins (C+E) supplementation in the prevention of preeclampsia: An RCT

**Published:** 2017-05

**Authors:** Milad Azami, Tayebe Azadi, Sepidezahra Farhang, Shoobo Rahmati, Khadijeh Pourtaghi

**Affiliations:** 1 *Student Research Committee, Ilam University of Medical Sciences, Ilam, Iran.*; 2 *Nursing and Midwifery Faculty, Ilam University of Medical Sciences, Ilam, Iran.*; 3 *Faculty of Medicine, Ilam University of Medical Sciences, Ilam, Iran.*

**Keywords:** Prevention, Preeclampsia, Calcium, Magnesium, Zinc, Vitamin D, Vitamin C, Vitamin E

## Abstract

**Background:**

Several studies have reported the uncertain role of multi-minerals and vitamins in the prevention of preeclampsia.

**Objective:**

The present study aims to investigate the effect of multimineral-vitamin D supplements (calcium, magnesium, zinc and Vitamin D) and vitamins (C+ E) in the prevention of preeclampsia.

**Materials and Methods:**

In this randomized clinical trial, 90 pregnant women were divided into three groups: group A received Ferrous sulfate (1 tablet/day) + one tablet of Claci-care multimineral-vitamin D containing 800mg calcium, 200mg magnesium, 8mg zinc and 400 IU Vitamin D3 per day; group B received Ferrous sulfate (1 tablet/day) + 250 mg vitamin C + 55 mg vitamin E; and the controls received only one Ferrous sulfate tablet daily.

**Results:**

The incidence of preeclampsia in group A was significantly lower than the control group (p=0.03), while there was no significant difference between group B and controls (p=0.50), as well as groups A and B (p=0.063). The incidence of neonatal complications in the group A was significantly lower than the control group (p=0.01), while there was no significant difference between group B and control (p=0.48).

**Conclusion:**

According to the results, calcium, magnesium, and zinc supplements have a significant effect on the prevention of preeclampsia. In addition, prescription of multimineral-vitamin D during pregnancy can be a low-cost and affordable way to reduce the incidence of preeclampsia in women who are at high risk of preeclampsia.

## Introduction

Preeclampsia (PE) is a leading cause of maternal and prenatal mortality, particularly in poor and developing countries (1). The prevalence of PE has been reported to be 5-7% of all pregnancies (2-4). PE is described by certain symptoms including: high blood pressure (≥140/90) after week 20 of pregnancy, and proteinuria ≥300 mg per 24 Hr. equal to +1 in urine dipstick (1, 5). PE affects mothers and fetuses’ health during pregnancy, therefore, PE prevention is highly crucial (1). The most serious complications of PE in mothers and fetuses’ health include increasing maternal mortality, thrombophilic disorders, edema, eclampsia, liver or kidney failure, stroke, cardiovascular diseases, intrauterine growth retardation, prematurity, and embryonic death (6-10).

Some studies indicate poor nutrition (especially protein, calcium (Ca), sodium, magnesium (Mg), and vitamins )A, C, E) as factors that exacerbate PE. Furthermore, in some researches diets including calcium supplements have been rejected regarding prevention of PE (11-13). Kanagal *et al* and Jain *et al* concluded that serum levels of multimineral-vitamin D (Zinc (Zn), Mg, Ca and vitamin D3) during pregnancy can affect the preeclampsia. However, this was not significant in the study of Vafaei *et al* (14-16). 

Considering the importance of PE in maternal and fetal health as well as controversial results of studies on the role of multimineral-vitamin D and vitamins in the prevention of PE, the present study aims to investigate the effect of multimineral-vitamin D supplementation (Zn, Mg, Ca and vitamin D3) and vitamins (C+E) in the prevention of PE.

## Materials and methods

**Study design**

In this randomized controlled clinical trial, 90 pregnant women who referred to Ilam Educational Center of Obstetrics and Gynecology, Iran in 2014, were divided into three groups (n=30). The sample size was determined based on 95% confidence interval, an alpha error of 5%, the power of 90%, and the prevalence of 5-7% PE (2-4). 

Our inclusion criteria included women with least one of the risk factors for PE [including chronic vascular disease, hydatidiform mole, multiparity, diabetes mellitus, thyroid disease, chronic hypertension, nulliparity, history of preeclampsia, maternal age >35 years, kidney disease, collagen vascular disease, antiphospholipid antibody syndrome, family history of preeclampsia, history of thrombophilia, and obesity (BMI >25)] older than 20 weeks of gestational age who have received ferrous sulfate according to prenatal care program. Women who had several changes in diet during the trial were excluded from the study.

After obtaining informed consent and explaining the purpose of the study, participants were randomly divided into 3 groups according to randomized selection: Group A received one Ferrous sulfate tablet (Rooz daru©, Iran) + one Claci-care multimineral-vitamin D tablet [(Vitane Pharma©, Germany) contained 800 mg Ca, 200mg Mg, 8mg Zn and 400 IU vitamin D3)] per day; Group B received one Ferrous sulfate tablet (Rooz daru©, Iran,) + 250 mg vitamin C and 55 mg vitamin E, and control group only received Ferrous sulfate daily.

The patients with gestational age of 20-28, 28-36, and >36 wk were visited monthly, once every 2 wk, and weekly, respectively, for evaluation of blood pressure and proteinuria in our Obstetrics and Gynecology Clinic. Diagnosis of PE was done by a gynecologist using the high blood pressure ≥140/90 after week 20 of pregnancy and proteinuria ≥300 mg per 24 Hrs. or equal to +1 in urine dipstick (1, 5).

**Ethical consideration**

This study was approved by the Ethics Committee of the Medical University of Ilam, Iran (EC/93/H223).

**Statistical analysis**

Data were analyzed using Statistical Package for the Social Sciences, version 17.0, SPSS Inc, Chicago, Illinois, USA (SPSS) as well as Student’s *t*-test, Chi-square, Fisher, and ANOVA test.

## Results

**Figure 1:**
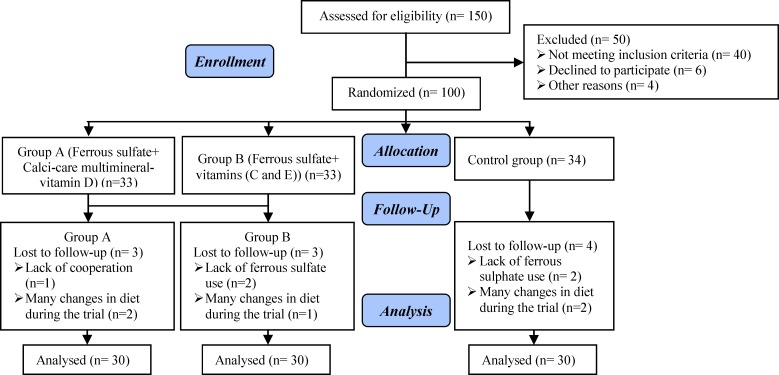
Follow-up Method Based on CONSORT 2010 Flow Diagram

100 pregnant women were randomly divided into three groups: Group A (n=33), group B (n=36) and control group (n=34). In the control group, four women (lack of ferrous sulphate use [n=2] and many changes in diet during the trial [n=2]) were excluded. The exclusions in the group A included three women (lack of cooperation [n=1] and many changes in diet during the trial [n=2]). The exclusions in the group B included three women (lack of ferrous sulfate use [n=2] and many changes in diet during the trial [n=1]). Finally, 90 women (Group A [n=30], group B [n=30] and control group [n=30]) completed the trial (Figure 1).

**Figure 2:**
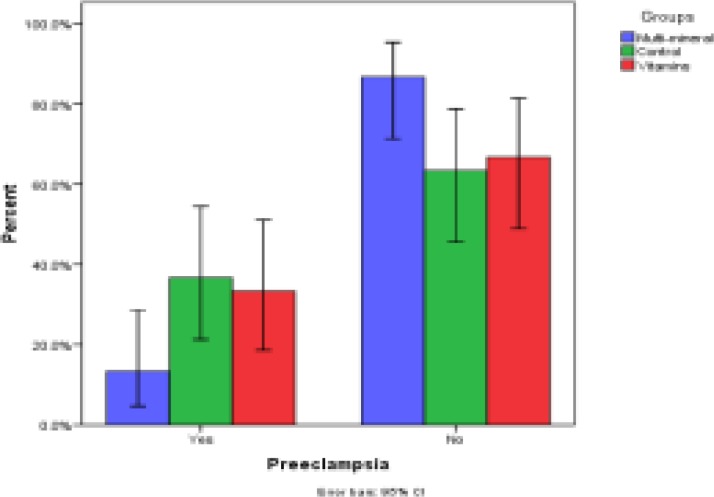
Comparison of the effect of multimineral-vitamin D (Zn, Mg, Ca and vitamin D3) and vitamins (C and E) supplementations in the prevention of preeclampsia

**Figure 3:**
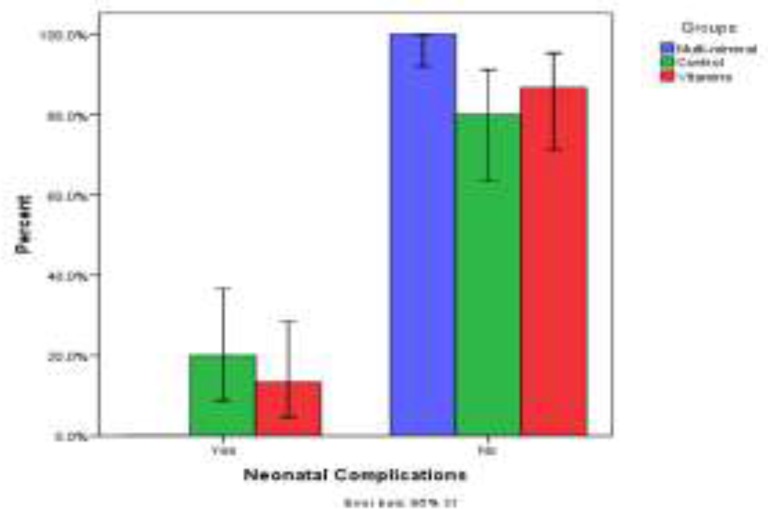
Comparison of the effect of multimineral-vitamin D (Zn, Mg, Ca and vitamin D3) and vitamins (C and E) supplementations in the neonatal complications

Overall, 90 pregnant women in three groups were included in this study. The mean age of study participants was 31.63±6.13 yr. The mean age of participants was not statistically different between groups A, B, and controls (33.03±6.49, 31.73±6.41, and 30.63±5.28 yr, respectively, P=0.18) Demographic characteristics are shown in table I. The incidence of preeclampsia in the group A was significantly lower than the controls (13.3% vs. 36.7%, p=0.03) but the incidence of preeclampsia in the group B was not different from the control group (33.3% vs. 36.7%, p=0.50) and also groups A and B (p=0.063) (Figure 2). Figure 3 shows the risk of neonatal complications in the A, and control groups. Group A had a signiﬁcantly reduced risk of neonatal complications (p=0.01), but this association in the group B was not statistically significant (p=0.48).

**Table I:** Demographic characteristics of participants in three groups

**Variables**			**Group A (n=30)**	**Group B (n=30)**	**Control (n=30)**	**Total**	**p-value**
Age (yr)*			33.03±6.49	31.73±6.41	30.13±5.28	31.63±6.13	0.188
Gestational age (at the delivery time) (wk)*			37.53±1.47	38.00±2.49	37.56±1.52	37.7±1.88	0.568
Level of education n(%)							
	Under diploma	2 (8.7)		8 (34.8)	13	23	0.004
	Diploma	20 (37)		20 (37)	14	54	
	Undergraduate	8 (61.5)		2 (15.4)	3	13	
Job n(%)							
	Housekeeper	21 (27.3)		26 (33.8)	30 (39)	77	0.004
	Employee	9 (69.2)		4 (30.8)	0 (0)	13	
Family monthly income n(%)							
	<0.5 million toman	0 (0)		0 (0)	4 (100)	4	0.002
	0.5-1 million toman	7 (21.2)		9 (27.3)	17 (51.5)	33	
	1-2 million toman	17 (38.6)		18 (40.9)	9 (20.5)	44	
	2-2.5 million toman	1 (50)		1 (50)	0 (0)	2	
	2.5-3 million toman	4 (100)		0 (0)	0 (0)	4	
	>3 million toman	1 (33.3)		2 (66.7)	0 (0)	3	
Residence n (%)							
	Urban	30 (46.9)		22 (34.4)	12 (18.8)	64	0.000
	Rural	0 (0)		8 (69.2)	18 (30.8)	26	
Type of delivery n(%)							
	Normal vaginal delivery	7 (20.6)		10 929.4)	17 (50)	34	0.024
	Cesarean section	23 (41.1)		20 (35.7)	13 (23.2)	56	

Chi-Square Tests for qualitative variables and ANOVA Test for quantitative variable

* Data presented as mean±SD.

## Discussion

This study showed that consumption of multimineral-vitamin D (Zn, Mg, Ca and vitamin D3) among pregnant women at risk of preeclampsia reduced the incidence of preeclampsia. In this regard, Jain and Kim in their study concluded that administration of multi-minerals (Ca, Mg, and Zn) during pregnancy can be effective in the prevention of preeclampsia (15, 17). Abedi *et al* in a study demonstrated that vitamin D levels in preeclampsia group was less than healthy subjects, suggesting that vitamin D is effective in prevention of preeclampsia (18). In addition, Kanagal *et al* found that serum Ca concentration was significantly lower in the preeclampsia group compared with healthy pregnant women (14). The results of these studies were consistent with our results. But Vafaei *et al* reported that the serum levels of Ca, Mg, and Zn in normotensive women were not significantly different from preeclamptic women (16).

Comparing the case group (E and C) with control group showed a percentage of 33.3% and 36.6% preeclampsia in pregnant women and this difference was not statistically significant (p=0.50). In this regard, Spinnato *et al* in a clinical trial showed that the incidence of preeclampsia was 13.8% in pregnant women who received vitamins E+C and 15.6% in women who received placebo, and this difference was not statistically significant (19).

Moreover, based on two systematic reviews by Polyzos and Salles *et al* showed that taking vitamins E and C does not reduce the risk of preeclampsia during pregnancy (20, 21). However, studies of Chappell *et al* and Zhang *et al* among women at risk of preeclampsia demonstrated that co-administration of vitamins E and C effected the prevention of pre-eclampsia (22, 23). According to another study, Bowen *et al* indicated that vitamin C reduced the risk of preeclampsia (24). Therefore, the results of these studies are not in line with the present study, which might be due to different sample size, different populations studied in various geographical areas, diversity in nutrition and diet and different races.

In this study, there was a significant reduction in the incidence of neonatal complications in the multimineral-vitamin D supplementation group, but this association in the vitamins supplementation group was not statistically significant. So, it seems that multimineral-vitamin D supplementation in pregnant women at the risk of preeclampsia decreased the risk of neonatal complications by reducing the incidence of preeclampsia. 

Furthermore, in the present study, no significant relationship was found between the mother’s obesity and incidence of preeclampsia (p=0.56) which was consistent with the experiment of Dabirieskoei *et al* (25). Furthermore, a systematic review and meta-analysis of 13 cohort studies showed that with increasing 5-7 kg/m^2^ of the mother’s weight, the risk of preeclampsia doubles, which, due to low sample size of our study, our results were not consistent with this meta-analysis (26). 

In this study, there was a significant reduction in the incidence of neonatal complications in the multimineral-vitamin D supplementation group. Furthermore, in a clinical trial by Asemi *et al* which analyzed the effect of multi-mineral and vitamin D supplement on pregnancy outcomes in pregnant women at risk for pre-eclampsia, showed that consumption of multi-minerals (Zn, Mg, and Ca) leads to an increase height, calcium, magnesium, zinc levels in newborns compared with the placebo group (27). Robinson and Ghomian studied the relationship between vitamin D deficiency, preeclampsia and fetal growth retardation and indicated that Vitamin D supplementation may improve the outcome of pregnancy to prevent or delay pre-eclampsia and fetal growth problems in the high-risk group (28, 29).

Overall, it seems that multimineral-vitamin D supplementation in pregnant women at the risk of preeclampsia decreased the risk of neonatal complications by reducing the incidence of preeclampsia. Moreover, the present study showed that association between vitamins E+C supplementation group and incidence of neonatal complications was not statistically significant.

In this regard, Polyzos *et al* in their systematic review demonstrated that consumption of vitamin C and vitamin E supplements during pregnancy cannot decrease the risk of fetal or neonatal death, preterm delivery and small size for gestational age (20). Rumbold in a review study regarding the effect of co-administration of vitamins E+C during pregnancy did not report a significant relationship with reduction of pre-eclampsia and other serious adverse effects of pregnancy (30). Moreover, Rumbold achieved similar results in another study (31). Rahmaniyan showed that consumption of vitamin C and vitamin E supplements reduces risk of low birth weight, but does not effect stillbirth and small size for gestational age (32).

**Limitation**

Limitations of the present study include not dividing preeclampsia into different severities. Therefore, we couldn’t investigate the relationship between supplementations and the severity of preeclampsia or gestational age. Moreover, considering failure to check nutritional information and diet in the patients because of lack of cooperation, it is recommended to be considered in future studies. 

## Conclusion

According to the results of this study, considering the significant effect of multi-minerals supplements (Ca, Mg, and Zn) in the prevention of preeclampsia and taking these multi-minerals during pregnancy can be a low-cost and affordable way to reduce the incidence of preeclampsia in women who are at high risk of preeclampsia. On the other hand, it was found that taking multi-minerals reduce the incidence of neonatal complications. Vitamins C and E supplementation had no effect on the prevention and complications of preeclampsia.
